# Endoscopic Ultrasound‐Guided Tissue Acquisition for Diagnosing Hepatic Malignant Lymphoma

**DOI:** 10.1002/ccr3.71927

**Published:** 2026-01-21

**Authors:** Yuichi Takano, Naoki Tamai, Jun Noda, Tetsushi Azami, Fumitaka Niiya, Masatsugu Nagahama

**Affiliations:** ^1^ Division of Gastroenterology, Department of Internal Medicine Showa Medical University Fujigaoka Hospital Yokohama Kanagawa Japan

**Keywords:** endo‐hepatology, endoscopic ultrasound, malignant lymphoma, tissue acquisition

## Abstract

A 62‐year‐old man with right upper quadrant pain and fever was found to have numerous hepatic masses. Endoscopic ultrasound‐guided tissue acquisition (EUS‐TA) using a 22‐gauge fine‐needle biopsy enabled diagnosis of high‐grade T‐cell lymphoma without adverse events. This case underscores the potential of EUS‐TA as a safe and effective alternative to percutaneous biopsy for hepatic malignant lymphoma.

## Key Clinical Message

- In recent years, the concept of “Endo‐Hepatology” has increased awareness of the clinical utility of EUS‐TA for liver diseases.
- Fine needle biopsy needles enable the diagnosis of hepatic malignant lymphoma in combination with immunohistochemistry.
- EUS‐TA may serve as an alternative to percutaneous biopsy in suspected cases.

## 1 Case Presentation

**FIGURE 1:**
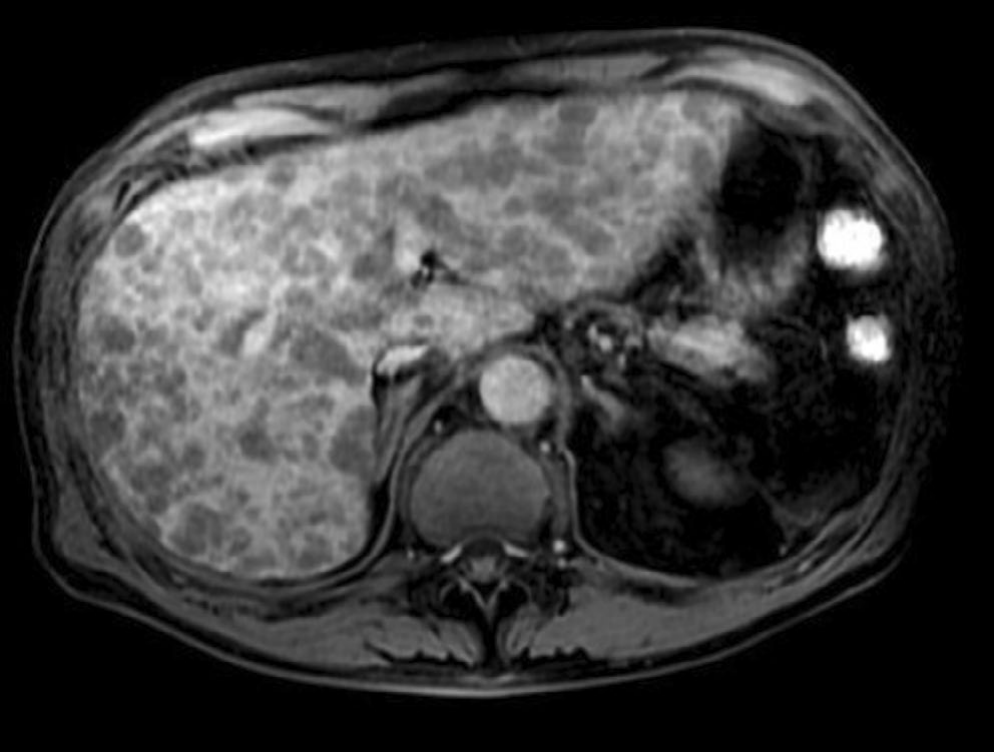
Contrast‐enhanced magnetic resonance imaging in the hepatobiliary phase demonstrates numerous small‐to‐medium‐sized, well‐defined hypointense nodules diffusely distributed throughout both hepatic lobes, without formation of a dominant mass. This multifocal distribution pattern is less suggestive of primary hepatic carcinoma and is more consistent with diffuse hepatic involvement by malignant lymphoma, while diffuse‐type metastatic liver tumors are also considered in the differential diagnosis.

**FIGURE 2:**
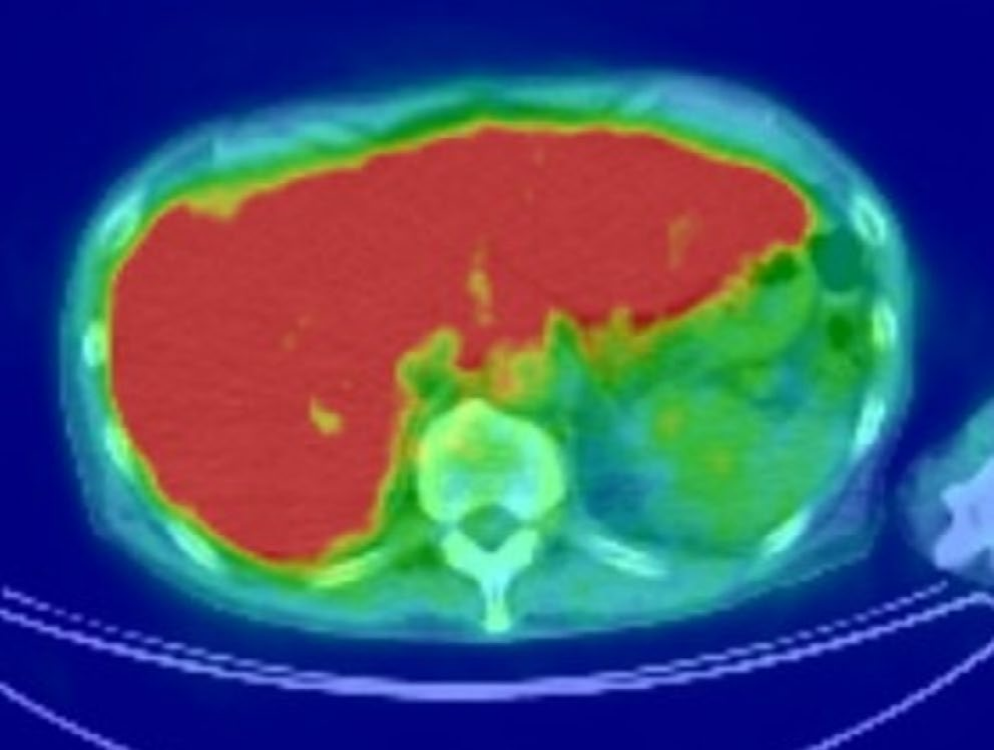
Positron emission tomography reveals diffuse uptake in the liver.

**FIGURE 3:**
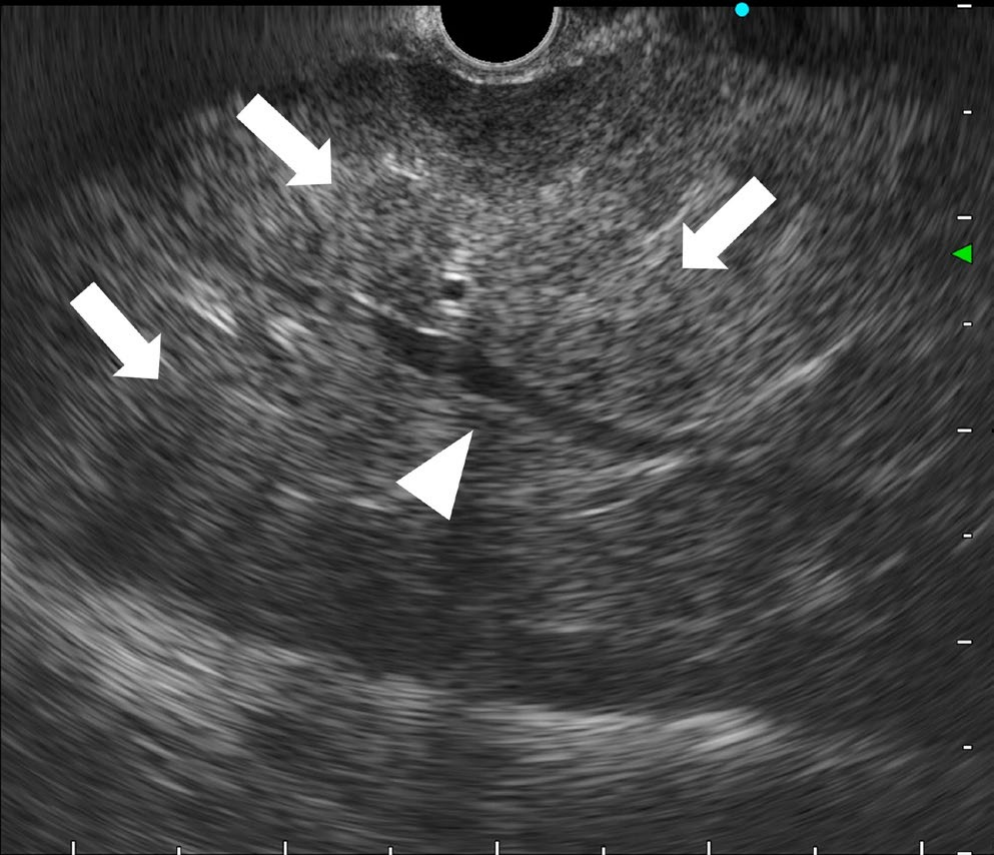
Endoscopic ultrasonography demonstrates multiple ill‐defined, hypoechoic hepatic masses diffusely distributed in the left hepatic lobe (arrow). The lesions show relatively homogeneous internal echoes without a distinct capsule, with preserved intrahepatic vascular structures (arrowhead), reflecting a compressive growth pattern characteristic of malignant lymphoma. A potential pitfall is a false‐negative result due to inadvertent sampling of normal hepatic parenchyma. Therefore, even in ill‐defined lesions, careful EUS observation is essential, and targeted biopsy is performed toward hypoechoic areas that are confidently considered to represent the lesion.

**FIGURE 4:**
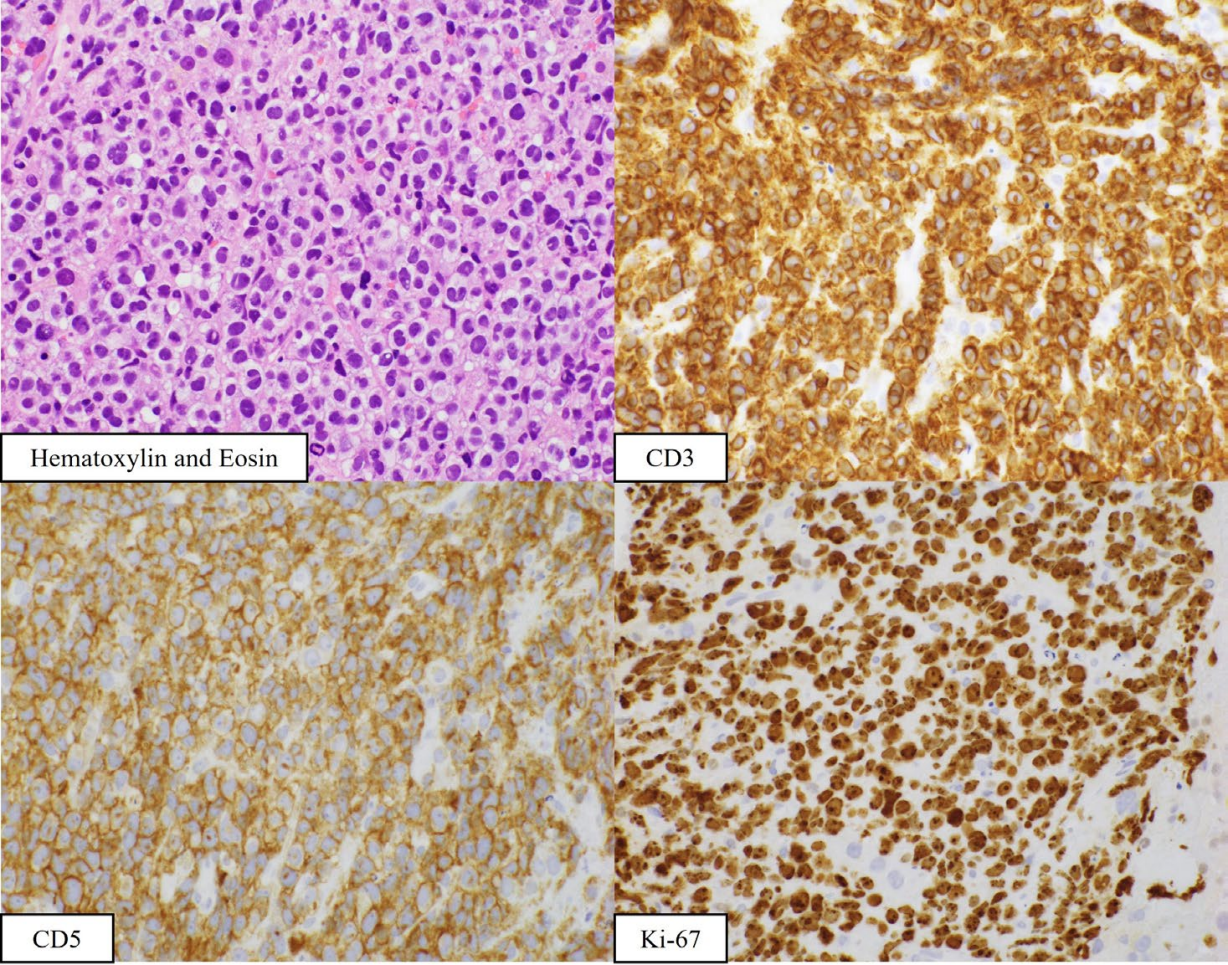
Histopathological analysis reveals atypical lymphoid cells. Immunohistochemistry shows CD3(+), CD5(+), CD10(−), CD20(−), CD56(−), CD79a(−), and Ki‐67 > 90%. A diagnosis of high‐grade T‐cell lymphoma is made.

A 62‐year‐old man presented with right upper quadrant abdominal pain and fever lasting for 2 weeks. Magnetic resonance imaging and positron emission tomography revealed numerous hepatic masses with diffuse fluorodeoxyglucose uptake (Figures 1 and 2). Serum soluble interleukin‐2 receptor levels were markedly elevated (204,882 U/mL), raising suspicion for malignant lymphoma. Endoscopic ultrasound (EUS) demonstrated multiple hypoechoic hepatic lesions in the left lobe with preserved intrahepatic vascular architecture (Figure 3). After confirming the absence of intervening vessels on Doppler imaging, the hypoechoic lesion was punctured transgastrically using a 22‐gauge fine needle biopsy (FNB) needle. Tissue acquisition was performed using the slow‐pull technique, and the presence of whitish core tissue was confirmed by macroscopic on‐site evaluation. Rapid on‐site evaluation was not performed. No procedure‐related adverse events were observed. Histopathology revealed atypical lymphoid cells. Immunohistochemical staining showed CD3(+), CD5(+), CD10(−), CD20(−), CD56(−), CD79a(−), and Ki‐67 > 90% (Figure 4). A diagnosis of high‐grade T‐cell lymphoma was established, and systemic chemotherapy was initiated.

## 2 Discussion

Percutaneous biopsy remains the gold standard for histopathological diagnosis of liver disease, especially in cases like malignant lymphoma, in which immunohistochemical evaluation is indispensable. However, patients with malignant lymphoma or leukemia have been reported to be at increased risk of post‐procedural bleeding, and although rare, fatal cases have occurred [1, 2]. In recent years, the concept of “Endo‐Hepatology” has gained traction, and the utility of EUS‐TA for liver diseases has been increasingly recognized. EUS‐TA for focal liver lesions has been shown to achieve diagnostic adequacy comparable to percutaneous biopsy [3].

In the diagnosis of malignant lymphoma, large‐bore needles such as 19‐gauge were traditionally used; however, the advent of fine‐needle biopsy (FNB) technology has enabled reliable histopathological and immunohistochemical evaluation even with 22‐gauge needles. EUS‐TA offers several advantages over percutaneous biopsy, including the use of thinner needles, patient immobility under sedation, and high‐resolution imaging that allows precise avoidance of vascular structures. These factors may reduce the risk of bleeding and enhance procedural safety.

## 3 Conclusions

This case highlights the potential utility of EUS‐TA as an alternative to percutaneous biopsy in patients with suspected hepatic malignant lymphoma.

## Author Contributions

**Yuichi Takano:** conceptualization, writing – original draft, writing – review and editing. **Naoki Tamai:** writing – review and editing. **Jun Noda:** writing – review and editing. **Tetsushi Azami:** writing – review and editing. **Fumitaka Niiya:** writing – review and editing. **Masatsugu Nagahama:** writing – review and editing.

## Ethics Statement

The authors have nothing to report.

## Consent

Written informed consent was obtained from the patient to publish this report in accordance with the journal's patient consent policy.

## Conflicts of Interest

The authors declare no conflicts of interest.

## Data Availability

The data that support the findings of this study are available from the corresponding author upon reasonable request.
